# Hidden in Plain Sight: Systemic Mastocytosis Manifesting as Isolated Hepatosplenomegaly in the Absence of Cutaneous and Classical Manifestations—A Case Report and Literature Review

**DOI:** 10.1002/ccr3.72960

**Published:** 2026-06-26

**Authors:** Muhammad Sadam Zeb, Ubaid Ullah Mian, Kashif Khan, Aiman Gulalay, Alishba Hameed, Alyan Malook, Essam Saeed, Suleman Khan, Muhammad Mujtaba, Kamil Ahmad Kamil

**Affiliations:** ^1^ Khyber Medical College Peshawar KPK Pakistan; ^2^ Quality Control Coordinator, Department of Research Naseer Teaching Hospital Peshawar KPK Pakistan; ^3^ Internal Medicine Department Nasrat Bashir Curative Hospital Kandahar Afghanistan

**Keywords:** hepatosplenomegaly, KIT D816V, leukocytosis, myeloproliferative neoplasm, SM‐AHN, systemic mastocytosis

## Abstract

Systemic mastocytosis (SM) is a rare clonal myeloproliferative neoplasm typically characterized by cutaneous lesions and mediator‐release symptoms. Presentations dominated by visceral organ involvement without skin findings are uncommon and pose a significant diagnostic challenge, often mimicking hematologic malignancies. We report a 50‐year‐old male presenting with generalized weakness, fatigue, and progressive abdominal distension. Examination revealed significant hepatosplenomegaly with no cutaneous manifestations. Investigations demonstrated severe leukocytosis (peak WBC 112.8 × 10^3^/μL) with a leucoerythroblastic picture, persistently elevated alkaline phosphatase, and anemia. Initial bone marrow morphology suggested chronic myeloid leukemia (CML), showing marked hypercellularity (95–100%) and granulocytic hyperplasia (M:E ratio 32:1). Cytogenetics revealed a normal male karyotype (46, XY), and BCR‐ABL1 testing was negative, excluding CML. Comprehensive molecular profiling identified a pathogenic KIT p.D816V mutation, confirming systemic mastocytosis with an associated hematologic neoplasm (SM‐AHN). The patient was treated with cladribine (40 mg over five days) followed by maintenance hydroxyurea (300 mg twice daily), with significant clinical improvement at eight‐week follow‐up. This case underscores that SM‐AHN can present with isolated hepatosplenomegaly and profound leukocytosis without cutaneous signs, and highlights the critical role of integrated molecular profiling, including KIT mutation analysis, in the diagnostic workup of atypical hematologic presentations.

## Key Clinical Message

Systemic mastocytosis can present with isolated hepatosplenomegaly and marked leukocytosis without skin lesions, mimicking BCR‐ABL1‐negative MPNs. While full WHO criteria require tryptase and CD25 IHC, KIT D816V mutation testing, often the only available assay in resource‐limited settings, provides a diagnostic anchor. SM‐AHN requires dual cytoreductive therapy targeting both mast cell and myeloid components.

## 1 Introduction

Mastocytosis encompasses a heterogeneous group of rare clonal myeloid disorders characterized by the abnormal proliferation and tissue accumulation of mast cells. The disease spectrum spans cutaneous mastocytosis, systemic mastocytosis (SM), and mast cell sarcoma [1, 2]. Cutaneous mastocytosis is predominantly confined to the skin and is more common in children, while SM is defined by extracutaneous organ involvement, typically confirmed through bone marrow examination [3, 4, 5]. The estimated prevalence of SM is approximately 1–2 per 10,000 adults, with an incidence of roughly 0.5–1 per 100,000 person‐years [6, 7]. Adult‐onset disease typically follows a persistent course and carries a greater risk of extracutaneous organ involvement compared with pediatric cases [7]. Epidemiologically, SM demonstrates a slight male predominance and arises predominantly as a sporadic condition; familial forms account for only approximately 4% of cases [8, 9].

Although hepatic and splenic involvement may be seen in up to 20%–30% of systemic cases, presentations characterized by isolated hepatosplenomegaly in the complete absence of cutaneous findings remain exceptionally uncommon [1, 3]. Most patients in published series exhibit skin lesions or mediator‐related symptoms, making visceral‐dominant, skin‐negative SM a diagnostic rarity. Clinically, SM may produce flushing, pruritus, abdominal pain, diarrhea, hypotension, syncope, fatigue, musculoskeletal pain, and constitutional symptoms [2, 10]. Anaphylaxis—particularly to Hymenoptera venom—and osteoporosis with atypical fractures are hallmark presentations that should raise clinical suspicion even in the absence of skin findings [10, 11]. The absence of dermatologic signs can substantially delay recognition, leading to misinterpretation as lymphoma, myeloproliferative neoplasms, or infiltrative disorders [2].

We describe a rare case of a 50‐year‐old male with SM‐AHN presenting without any cutaneous manifestations, whose initial clinical and morphological picture closely mimicked BCR‐ABL1‐negative chronic myeloid leukemia. This case illustrates the diagnostic pitfalls of skin‐negative SM and emphasizes the indispensable role of molecular testing in establishing a timely and accurate diagnosis [12, 13].

## 2 Case Presentation and Management

### 2.1 Clinical History

A 50‐year‐old male presented to the medical outpatient department with complaints of generalized weakness, fatigue, diffuse body aches, and progressive abdominal swelling of several months' duration. He explicitly denied fever, unintentional weight loss, night sweats, bleeding tendency, flushing episodes, syncope, diarrhea, or anaphylactic reactions. Pertinently, there was no history of pruritus, rashes, or pigmentary skin lesions. He had no significant past medical history, no known chronic illness, and a negative family history of systemic or autoimmune disease.

### 2.2 Physical Examination

**FIGURE 1:**
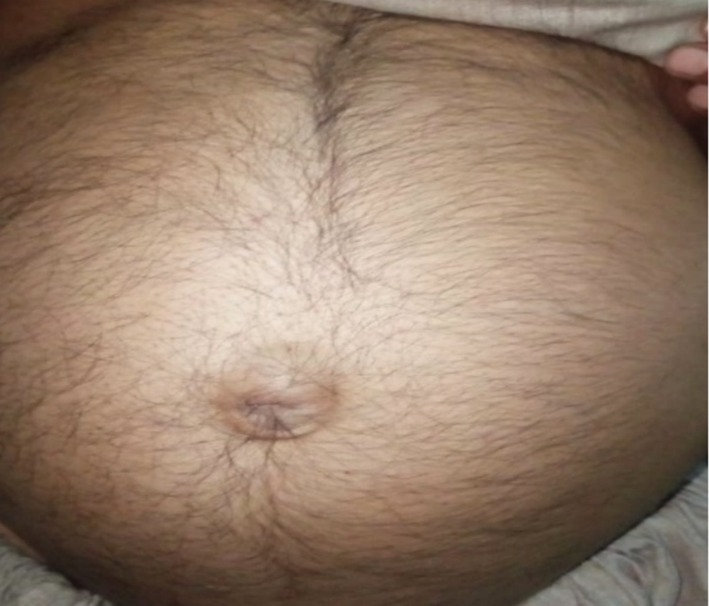
Anterior view of a distended abdomen without any cutaneous manifestations. (Clinical photograph demonstrating significant abdominal distension secondary to hepatosplenomegaly, with a complete absence of the urticaria pigmentosa or other dermatological signs typically associated with mastocytosis).

**FIGURE 2:**
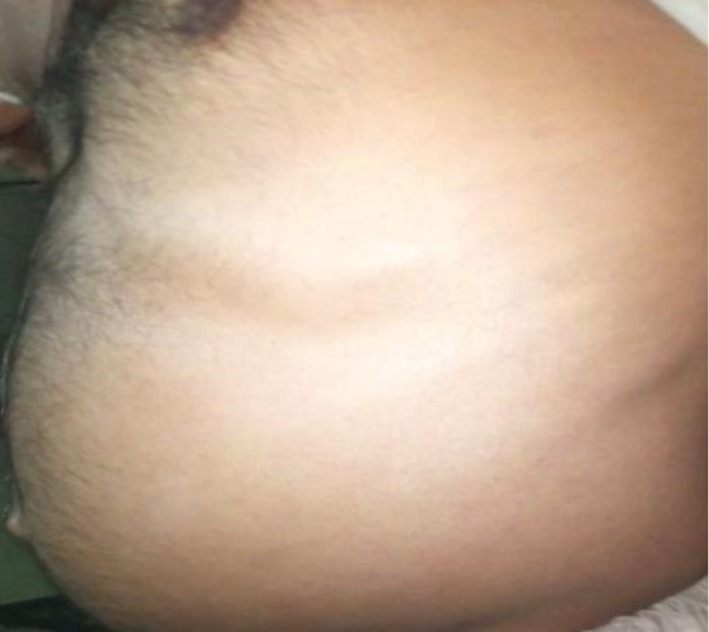
Lateral View of distended abdomen without any cutaneous lesions. (Lateral clinical photograph further illustrating the pronounced abdominal protrusion due to organomegaly, confirming the lack of cutaneous stigmata on the trunk).

**FIGURE 3:**
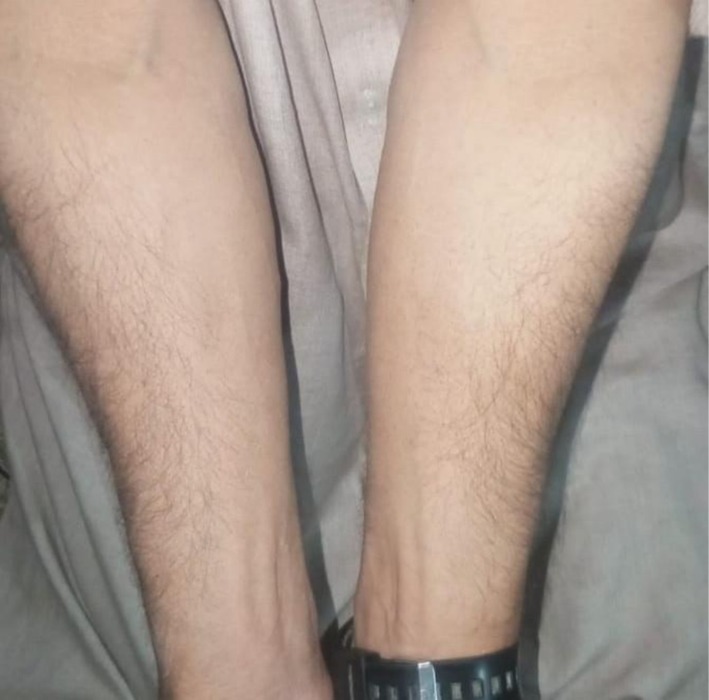
Anterior aspect of arms showing no cutaneous manifestations of the disease. (Close‐up photograph of the patient's upper extremities showing clear skin. A comprehensive dermatologic examination of the trunk, upper thighs, and posterior surfaces (including buttocks and back) also revealed no urticaria pigmentosa, maculopapular lesions, telangiectasias, or pigmentary changes. The absence of skin findings was confirmed on complete skin examination).

The patient appeared pale but was hemodynamically stable. No peripheral lymphadenopathy was identified. Abdominal examination revealed visible distension with palpable hepatomegaly and splenomegaly. A thorough dermatological survey—including the trunk, extremities, palms, and soles—disclosed no evidence of urticaria pigmentosa, maculopapular lesions, telangiectasias, or other cutaneous stigmata of mastocytosis. The complete absence of skin findings was confirmed on systematic full‐body skin examination (Figures 1, 2, 3).

### 2.3 Investigations

Serial hematological, hepatic, and renal investigations were performed throughout the clinical course and are consolidated in Table 1. On presentation, complete blood count demonstrated significant leukocytosis (WBC 49.3 × 10^3^/μL), which escalated to a peak of 112.8 × 10^3^/μL at follow‐up, accompanied by progressive anemia and preserved platelet counts. Peripheral blood smear revealed a left‐shifted myeloid series with promyelocytes (9%), myelocytes (19%), metamyelocytes (9%), and approximately 1%–2% blasts, alongside a leucoerythroblastic picture with anisocytosis, poikilocytosis, macrocytosis, polychromasia, and nucleated red blood cells.

**TABLE 1:** Serial laboratory investigations (CBC, LFTs, and RFTs with electrolytes) throughout the treatment course.

Date	Complete Blood Count (CBC)									Liver Function Tests (LFTs)				Renal Function & Electrolytes				
	WBC ×10^3^/μL	HGB g/dL	HCT %	PLT ×10^3^/μL	MCV fL	Neut %	Lymph %	Mono %	Eos %	ALT U/L	ALP U/L	T.Bili mg/dL	Albumin g/dL	Urea mg/dL	Creat mg/dL	Na mmol/L	K mmol/L	Ca mg/dL
13‐Apr‐25	49.3 ↑	14	42	171	88	50	40	5 ↑	4	47 ↑	100	0.6	4.0	30	0.8	140	4.0	9.0
17‐Apr‐25	7.5	14	42	200	88	60	30	5	2	31	346 ↑	0.8	4.2	30	0.6	140	4.0	9.0
24‐Apr‐25	71.9 ↑	11.4	44	191	101.4 ↑	60	5 ↓	5	1	—	—	—	—	—	—	—	—	—
23‐May‐25	22.7 ↑	11.3 ↓	35.0 ↓	195	92.3	80.8 ↑	9.8 ↓	5	2	28.4	100	0.4	4.0	33.4	0.77	140	4.0	9.0
3‐Jun‐25	41.0 ↑	10.1 ↓	34.4	161	93.7	71.6	6.3	20.5 ↑	2	18.7	100	0.33	4.0	27.1	0.74	140	4.0	9.0
17‐Jun‐25	112.8 ↑	10.3 ↓	32.8 ↓	167	89.7	68.8	10	19.7	2	—	—	—	—	—	—	—	—	—
25‐Jun‐25	—	—	—	—	—	—	—	—	—	19.5	353 ↑	0.22	3.16 ↓	23.7	0.81	141.4	4.22	7.77 ↓
27‐Jun‐25	—	—	—	—	—	—	—	—	—	18.3	294 ↑	0.2	3.19 ↓	42	0.79	139	3.2 ↓	9.0
28‐Jun‐25	—	—	—	—	—	—	—	—	—	13.7	255 ↑	0.19	4.0	39.6	0.72	140	4.0	9.0
30‐Jun‐25	—	—	—	—	—	—	—	—	—	15.9	236 ↑	0.26	4.0	42	0.61	138	3.7	9.13
20‐Aug‐25	57.9 ↑	9.5 ↓	40	169	90	86 ↑	8 ↓	3	3	51 ↑	174	0.6	4.0	33	1.0	140	4.0	9.0

*Note:* Reference Ranges: WBC 4–11 | HGB 11.5–17.5 | HCT 36%–54% | PLT 150–450 | MCV 76–96 | Neut 40%–75% | Lymph 20%–45% | Mono 1%–10% | ALT ≤ 45 U/L | ALP ≤ 305 U/L | T.Bili ≤ 1.0 mg/dL | Albumin 3.5–5.4 g/dL | Urea 10–50 | Creat 0.5–1.5 | Na 135–150 | K 3.5–5.1 | Ca 8.0–10.0 mg/dL. ↑ = above normal, ↓ = below normal. ↑ Above normal, ↓ Below normal. Dates 24‐Apr and 17‐Jun are CBC‐only; 25‐Jun onward are chemistry‐only time points. Rows without CBC or chemistry data indicate that the respective panel was not collected at that visit.

Biochemical profiling demonstrated persistently elevated alkaline phosphatase (ALP) with only mild intermittent transaminase elevation. Bilirubin remained within normal limits throughout, while albumin was mildly reduced on follow‐up assessments, consistent with hepatic infiltrative disease rather than frank hepatocellular dysfunction. Renal function was well preserved. Notable electrolyte disturbances included transient hypocalcemia and isolated hypokalemia on two occasions, likely reflecting metabolic disturbances associated with high cell turnover.

**FIGURE 4:**
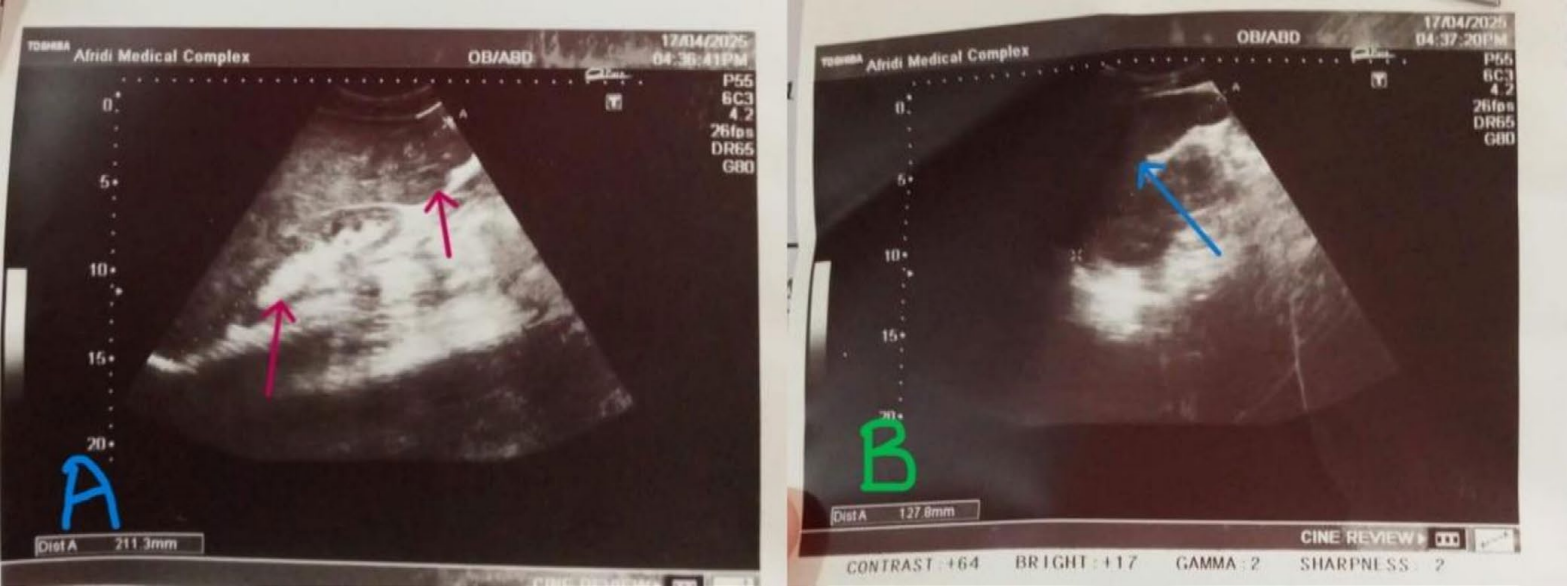
Ultrasonography of the abdomen, demonstrating hepatosplenomegaly. Ultrasound image revealing significant hepatomegaly (Figure 5A) (liver span 21.1 cm) with homogenous parenchymal echotexture. The spleen (Figure 5B) measures at the upper limit of normal (12.7 cm).

Viral serologies for hepatitis B and C on the day of presentation were negative, excluding viral hepatitis as a contributing cause. Abdominal ultrasonography (Figure 4) confirmed hepatomegaly (liver span 21.1 cm) with a spleen measuring at the upper limit of normal (12.7 cm).

### 2.4 Bone Marrow and Molecular Studies

Bone marrow trephine biopsy (24‐Apr‐25) revealed markedly hypercellular marrow (95–100%) with striking granulocytic hyperplasia, a myeloid‐to‐erythroid ratio of 32:1 (reference ~3:1), relative erythropoietic suppression, approximately 2% blasts, and minimal reticulin fibrosis (MF‐0/1). The initial pathological impression was chronic myeloid leukemia in chronic phase. However, on critical re‐review, scattered atypical mast cells with spindle‐shaped morphology, oval nuclei, and fine cytoplasmic granulation were identified, with clusters of > 15 mast cells in paratrabecular locations. Immunohistochemistry for CD117 and tryptase demonstrated strong cytoplasmic positivity, confirming mast cell lineage, while CD25 staining showed aberrant expression on neoplastic mast cells, fulfilling an additional minor WHO diagnostic criterion.

**FIGURE 5:**
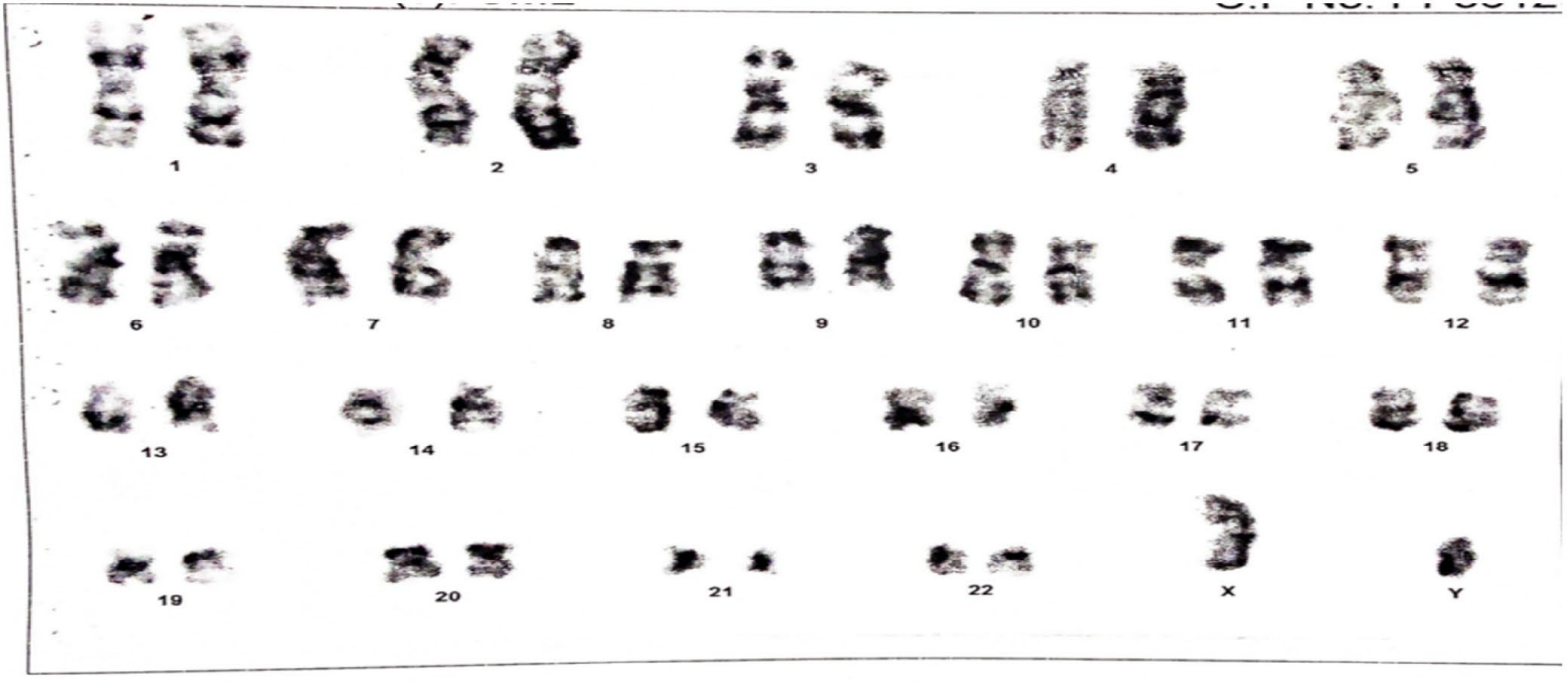
Cytogenetics/Karyotype Analysis.

Conventional cytogenetics (2‐May‐25) demonstrated a normal male karyotype (46, XY) with no Philadelphia chromosome on representative G‐banding (Figure 5). Molecular PCR for BCR‐ABL1 transcript was negative, definitively excluding CML. An extended MPN mutation panel was likewise negative for JAK2 V617F, CALR, and MPL mutations. Crucially, molecular analysis of KIT exon 17 identified a pathogenic p.D816V mutation, which—in concert with the morphological, immunohistochemical, and clinical findings—confirmed the diagnosis of systemic mastocytosis with an associated hematologic neoplasm of MPN‐like type (SM‐AHN).

### 2.5 Differential Diagnosis

The differential diagnosis at presentation encompassed BCR‐ABL1‐positive CML in chronic phase, BCR‐ABL1‐negative myeloproliferative neoplasms (polycythemia vera, essential thrombocythemia, primary myelofibrosis), myelodysplastic/myeloproliferative overlap syndromes, and infiltrative bone marrow disorders, including lymphoma. Systemic mastocytosis was introduced into the differential following the failure of the morphological picture to conform to any single MPN subtype and the negative molecular screen for classical MPN drivers. The diagnostic odyssey culminated in KIT D816V identification, resolving what had been a diagnostically ambiguous BCR‐ABL1‐negative leukocytosis with hepatosplenomegaly.

### 2.6 Treatment and Clinical Outcome

Following confirmation of SM‐AHN, the patient was initiated on cladribine (2‐chlorodeoxyadenosine) at a cumulative dose of 40 mg administered over five consecutive days (8 mg/day intravenously). The regimen was well tolerated, with mild heartburn as the sole adverse effect, managed successfully with omeprazole syrup. The patient was monitored in‐hospital for eight days and subsequently discharged on maintenance hydroxyurea 300 mg twice daily. He was enrolled in regular hematology outpatient follow‐up.

At the eight‐week post‐cladribine review, the patient demonstrated substantial clinical improvement. Fatigue and generalized weakness had largely resolved, and abdominal distension had decreased perceptibly. Physical examination confirmed a marked reduction in hepatosplenomegaly. Serial blood counts showed a progressive decline in leukocytosis (WBC 57.9 × 10^3^/μL at 20‐Aug‐25 vs. a peak of 112.8 × 10^3^/μL), although persistent leukocytosis indicated ongoing disease activity. The patient remained clinically stable on hydroxyurea, with no treatment‐related adverse effects. Repeat bone marrow biopsy and serum tryptase measurement were deferred due to resource limitations and patient preference for clinical monitoring.

**FIGURE 6:**
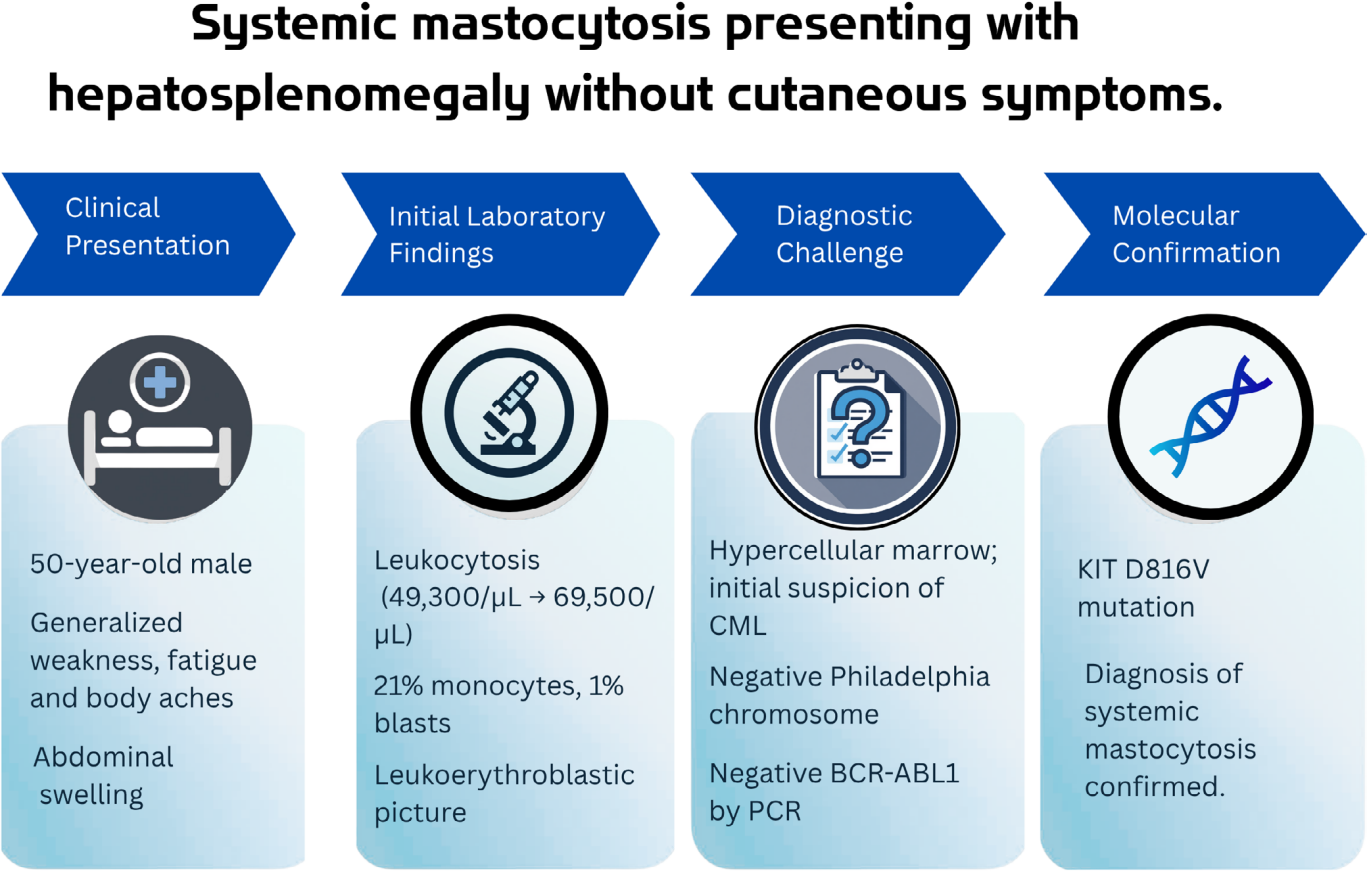
Diagrammatic representation of case management. Flowchart illustrating the diagnostic and therapeutic journey of a 50‐year‐old male presenting with unexplained leukocytosis and hepatosplenomegaly without cutaneous symptoms. The sequence outlines the initial laboratory findings, the diagnostic challenge of ruling out CML, molecular confirmation of the *KIT* D816V mutation, and final management, highlighting the clinical importance of early molecular testing in atypical systemic mastocytosis.

The following Figure 6 illustrates the diagnostic and therapeutic journey for a patient presenting with unexplained leukocytosis and hepatomegaly, culminating in the diagnosis of *KIT* D816V‐positive Systemic Mastocytosis with an Associated Myeloproliferative Neoplasm (SM‐AHN) and its subsequent management.

## 3 Discussion

Systemic mastocytosis is a clonal hematologic disorder driven by the neoplastic expansion of aberrant mast cells within one or more organ systems. The defining pathological hallmark is the formation of multifocal aggregates of atypical mast cells in affected tissues [12, 13]. Mast cells are long‐lived cells containing cytoplasmic granules rich in histamine, heparin, tryptase, and various cytokines; their growth and survival are critically dependent on stem cell factor (SCF) signaling through the KIT receptor [10, 14]. In adult‐onset SM, the constitutively activating KIT p.D816V mutation in exon 17 is identified in approximately 80%–90% of cases and drives autonomous mast cell proliferation and multi‐organ infiltration, as was confirmed in the present case [6, 15, 16].

The characteristic skin lesion of SM—urticaria pigmentosa (maculopapular cutaneous mastocytosis)—is present in the majority of patients and frequently provides the initial clinical clue [2]. However, SM can rarely present without cutaneous involvement [11, 17]. When skin lesions are absent, the diagnosis is substantially more challenging, and visceral organ findings dominate the clinical picture. Our patient exemplifies this rare phenotype: Hepatosplenomegaly was the exclusive clinical finding, while the profound leukocytosis and leucoerythroblastic blood picture compelled an initial working diagnosis of CML. This morphological mimicry of myeloproliferative disease is well recognized in the SM‐AHN subtype, wherein a concurrent clonal myeloid neoplasm produces hematological features that can entirely overshadow the mast cell component [12, 18].

Hepatic involvement in SM has been well described, typically manifesting as portal fibrosis, elevated ALP and γ‐glutamyl transpeptidase, and, in advanced cases, portal hypertension or hepatic failure [10, 11]. Our patient exhibited the characteristic biochemical signature of hepatic mast cell infiltration—disproportionate ALP elevation with minimal transaminitis and normal bilirubin—consistent with the infiltrative rather than hepatocellular pattern of liver disease. Notably, despite significant hepatosplenomegaly and elevated ALP, the patient did not develop ascites or portal hypertension during the observation period, distinguishing this presentation from several previously reported cases (Table 2) [14, 15]. Gastrointestinal involvement, reported in 70%–80% of SM patients, was also clinically absent here, though subclinical disease cannot be excluded without endoscopic and histological assessment [13, 19].

**TABLE 2:** Published case reports of systemic mastocytosis presenting with hepatosplenomegaly in the absence of cutaneous lesions.

Authors & Year	Title/Subtype	Presentation & Organs Involved	Treatment Strategy
M. Akin et al. [1]	SM with portal hypertension	Asymptomatic hepatosplenomegaly; portal hypertension, esophageal varices; no skin lesions. Liver (fibrosis, mast cell infiltration), spleen, and bone marrow.	Variceal banding; H1/H2 antihistamines; cromolyn sodium; interferon‐alpha considered.
K. S. Sujathan et al. (2014)	SM with portal HTN & hepatosplenomegaly	Weakness, abdominal distension; massive hepatosplenomegaly, ascites, portal hypertension; no urticaria pigmentosa. Liver (fibrosis), spleen, and bone marrow.	Diuretics for ascites; supportive care; specific SM therapy not detailed.
A. K. Ozdemir et al. (2011)	SM with skeletal involvement	Bone pain, fatigue, hepatosplenomegaly, widespread osteosclerotic lesions; no cutaneous findings. Bone, liver, spleen, and bone marrow.	Bisphosphonates; analgesics; mast cell stabilizers/antihistamines.
M. L. V. D. M. L. M. et al. (2008)	Indolent SM with hepatic involvement	Incidental hepatosplenomegaly; mild liver enzyme elevation; no skin or mediator symptoms. Liver, spleen, bone marrow (indolent).	Observation: Regular monitoring of liver/spleen size and tryptase levels.
S. Jain et al. (2018)	Smoldering SM	Early satiety, weight loss, abdominal fullness; massive splenomegaly, moderate hepatomegaly; no cutaneous signs. Spleen, liver, and bone marrow.	Cladribine (2‐CdA) to reduce mast cell burden; symptomatic management.
R. K. G. et al. (2016)	SM‐AHN (MDS) with hepatic failure	Rapid‐onset jaundice and hepatic failure; hepatosplenomegaly; no skin lesions. AHN was myelodysplastic syndrome. Liver (fulminant), spleen, and bone marrow.	Treatment directed at AHN/MDS; supportive care for hepatic failure. Poor prognosis.
Present Case (2025)	SM‐AHN (MPN‐like) – KIT D816V	Hepatosplenomegaly, profound leukocytosis (peak WBC 112.8 × 10^3^/μL), leucoerythroblastic picture; no skin lesions. Mimicked BCR‐ABL1‐negative MPN. Liver, spleen, and bone marrow.	Cladribine 40 mg over 5 days → maintenance hydroxyurea 300 mg BD. Dual cytoreductive targeting of mast cells and myeloid components.

*Note:* Comparative literature review contextualizing the present case within the spectrum of non‐cutaneous SM presentations.

The diagnostic workup in this case was complicated by the resource‐limited setting in which it was conducted. Complete WHO 2022 criteria for SM require: (1) a major criterion—multifocal dense mast cell aggregates of ≥ 15 cells per cluster in bone marrow or extracutaneous tissue; and (2) at least one of four minor criteria: (a) > 25% spindle‐shaped or atypical mast cells, (b) KIT codon 816 mutation, (c) aberrant CD2 and/or CD25 expression on mast cells, and (d) serum tryptase persistently > 20 ng/mL [20]. In our case, serum tryptase measurement was unavailable, and immunohistochemistry for CD25/CD2 was not performed initially. Despite this, the available evidence—KIT p.D816V positivity, absence of BCR‐ABL1/JAK2/CALR/MPL mutations, compatible bone marrow morphology with retrospective identification of mast cell clusters on re‐review, and the characteristic clinical triad of hepatosplenomegaly, leukocytosis, and absent skin lesions—converges strongly on SM‐AHN [18]. European Competence Network on Mastocytosis (ECNM) guidance acknowledges that KIT D816V detection with compatible clinical and morphological features may serve as a diagnostic anchor when full phenotypic assessment is not feasible [19, 20].

The treatment of SM‐AHN requires a dual cytoreductive strategy addressing both the mast cell clone and the associated myeloid neoplasm [12]. Cladribine (2‐CdA) has demonstrated efficacy across all SM subtypes, with published series reporting an overall response rate of approximately 55% at doses of 5 mg/m^2^/day for five days, with comparable response rates in indolent SM (56%), aggressive SM (50%), and SM‐AHN (55%).^21^ Our patient received cladribine at a cumulative dose of 40 mg over five days, consistent with established dosing practice, and achieved significant clinical improvement. Hydroxyurea was selected for maintenance cytoreduction of the myeloid AHN component, as described in prior SM‐AHN cohorts, at a dose of 300 mg twice daily [21]. Midostaurin, a KIT‐directed kinase inhibitor approved for advanced SM, and interferon‐alpha represent alternative or additional options not utilized in this case due to resource constraints and the favorable initial treatment response [12]. The sequential use of cladribine followed by maintenance hydroxyurea, in our case, adheres to documented cytoreductive strategies in SM‐AHN and is supported by the clinical outcome observed.

Comparing the present case with the literature (Table 2), a consistent theme emerges: SM presenting without skin lesions tends to occur in older adults, carries a greater burden of visceral organ involvement, and is frequently diagnosed with delay due to the absence of the classic cutaneous prompt. Prior reports have highlighted portal hypertension, skeletal disease, and gastrointestinal‐dominant presentations as alternative visceral phenotypes. The present case is distinctive in the degree of leukocytosis and the leucoerythroblastic blood picture, which produced a near‐complete morphological simulation of BCR‐ABL1‐negative MPN and necessitated dual‐modality cytoreductive therapy rather than the organ‐directed or symptomatic approaches sufficient in earlier reports.

## 4 Conclusion

This case illustrates that systemic mastocytosis—particularly the SM‐AHN subtype—can present with isolated hepatosplenomegaly and profound leukocytosis entirely devoid of cutaneous signs, closely mimicking BCR‐ABL1‐negative myeloproliferative neoplasms. In any patient with unexplained leukocytosis, hepatosplenomegaly, and negative BCR‐ABL1/JAK2/CALR/MPL testing, KIT D816V mutation analysis should be performed as an integral component of the diagnostic algorithm. In resource‐limited settings, KIT mutation positivity combined with compatible morphological and clinical features may serve as a sufficient diagnostic anchor to guide therapeutic decision‐making. SM‐AHN requires dual cytoreductive therapy directed at both the mast cell and myeloid components; in the present case, cladribine followed by maintenance hydroxyurea produced significant clinical benefit. Heightened awareness of this atypical, non‐cutaneous phenotype is essential to prevent diagnostic delay and ensure timely initiation of targeted therapy.

## 5 Limitations

Complete WHO 2022 diagnostic criteria were not formally fulfilled, as serum tryptase measurement, CD25/CD2 immunohistochemistry, and dedicated mast cell stains (CD117, tryptase) were unavailable in this resource‐limited setting. Quantitative assessment of spindle‐shaped mast cells was not reliably performed on archived material. Follow‐up was limited to eight weeks post‐cladribine, and neither repeat bone marrow biopsy nor tryptase monitoring was performed. Despite these constraints, the molecular‐clinical synthesis strongly supports the SM‐AHN diagnosis and preserves the educational value of this report.

## Author Contributions

**Muhammad Sadam Zeb:** conceptualization, data curation, formal analysis, investigation, methodology, resources, software, validation, writing – original draft, writing – review and editing. **Ubaid Ullah Mian:** conceptualization, data curation, formal analysis, investigation, methodology, project administration, resources, software, visualization, writing – original draft, writing – review and editing. **Kashif Khan:** formal analysis, methodology, resources, software, visualization, writing – original draft, writing – review and editing. **Aiman Gulalay:** conceptualization, investigation, methodology, project administration, software, validation, visualization, writing – original draft, writing – review and editing. **Alishba Hameed:** conceptualization, formal analysis, methodology, resources, validation, visualization, writing – original draft, writing – review and editing. **Alyan Malook:** conceptualization, formal analysis, project administration, resources, software, writing – original draft, writing – review and editing. **Essam Saeed:** data curation, methodology, project administration, resources, validation, visualization, writing – original draft, writing – review and editing. **Suleman Khan:** methodology, resources, software, supervision, validation, writing – original draft, writing – review and editing. **Muhammad Mujtaba:** conceptualization, methodology, project administration, supervision, writing – original draft, writing – review and editing. **Kamil Ahmad Kamil:** conceptualization, funding acquisition, investigation, methodology, project administration, resources, visualization, writing – original draft, writing – review and editing.

## Funding

The authors have nothing to report.

## Ethics Statement

The authors have nothing to report.

## Consent

Written informed consent was obtained from the patient and/or their legal guardian for the publication of this case report, including relevant clinical details and any accompanying images.

## Conflicts of Interest

The authors declare no conflicts of interest.

## Data Availability

The data used in this case report were obtained from a patient who presented to our hospital. All referenced studies and background information are publicly available on databases such as PubMed and Google Scholar. No additional datasets were generated or analyzed for this study.
